# Implementing a nutrition education intervention in Eastern Norwegian Kindergartens: barriers and facilitators

**DOI:** 10.1186/s40795-024-00908-z

**Published:** 2024-07-24

**Authors:** Caroline Løvik Brandvik, Biljana Meshkovska, Gry Irene Granli Schultz, Lisa Garnweidner-Holme

**Affiliations:** 1 Department of Nursing and Health Promotion, Faculty of Health Sciences, Oslo Metropolitan University, Post Box 4, St.Olavs Plass, 0130 Norway; 2 Department of Nutrition, Institute of Basic Medical Sciences, University of Oslo, Post Box 1072, Blindern, Oslo, 0316 Norway; 3 Centre for Global Health, Sustainable Health Unit, University of Oslo, Post Box 1072, Blindern, 0316 Norway; 4 Nes Municipality, Rådhusgata 2, Årnes, 2150 Norway

**Keywords:** Experiences, Implementation, Intervention, Kindergarten, Healthy eating

## Abstract

**Background:**

Implementations to improve healthy eating in kindergartens may play a pivotal role in shaping children’s dietary behaviors. There is limited research on the implementation and key implementation determinants (barriers and facilitators) of interventions in kindergarten settings. The aim of this study was to explore kindergarten staff members’ experiences with the implementation of a nutrition education intervention to identify implementation barriers and facilitators.

**Methods:**

We interviewed 12 employees from five different kindergartens in an Eastern Norwegian municipality between 2019 until 2020. The individual interviews were guided by the consolidated framework for implementation research. The interviews were recorded, transcribed verbatim, and analyzed inductively, inspired by Braun and Clarke’s reflexive thematic analysis.

**Results:**

Implementation facilitators were satisfactory planning and presentation execution, including tailoring to kindergarten and staff needs, food and meals being a kindergarten/staff priority, and confidence-building of staff. Barriers included unsatisfactory planning and presentation execution, the presentation as a one-time event, non-tailoring to kindergarten and staff needs, and kindergartens/staff not prioritizing food and meals.

**Conclusions:**

When developing and implementing similar kindergarten interventions, the following should be considered: a participatory approach, active engagement of staff, the physical learning environment, and the frequency of opportunities to revisit topics.

**Supplementary Information:**

The online version contains supplementary material available at 10.1186/s40795-024-00908-z.

## Introduction

The first years of life are characterized by rapid physical growth, and establishing healthy eating behaviors can serve as a solid foundation for future habits [1]. In Norway, 93.4% of children aged 1–5 years are in kindergarten. Kindergarten play a pivotal role in shaping children’s dietary behaviors due to the amount of time they spend there and the contextual environment in which these behaviors are developed [2]. In 2011, it was found that most Norwegian kindergartens facilitated three meals a day, with breakfast often brought from home (67%) and lunch (84%) and an afternoon meal (53%) provided by the kindergartens [3]. Given the duration of five years and receiving three meals a day, children consume 3,000 meals while in kindergarten [4].Thus, kindergarten is an important setting for public health interventions aimed at healthy eating [5, 6]. Previous reviews have reported that kindergarten policies and practices may be associated with a child’s improved dietary intake in this setting [7]. This includes kindergarten staff members’ feeding practices [8], increased availability and exposure to healthier options, increased knowledge of kindergarten staff and children through healthy eating education interventions, parental/carer involvement, and supportive healthy eating policies [2, 9].

The Norwegian guidelines for food and meals in kindergarten aim to ensure healthy food options in kindergartens nationwide [3]. The guidelines relate to the implementation of meals (e.g., duration, supervision, and physical and social facilitation), the nutritional quality of food and drink offered, food safety and hygiene, food allergies and intolerances, and environmental considerations. Well-implemented guidelines can potentially improve practices and contribute to equalizing social differences in health and diet among children nationwide [10]. However, the extent to which the national guidelines from 2018 are being implemented is unknown [11]. The Norwegian Institute of Public Health’s food mapping [3] and a more recent study of food and meal practices in a sample of Norwegian kindergartens from 2021 [12] emphasize that there is still room for improvement in food provided by kindergartens as well as increased knowledge, awareness, and active use of the guidelines. However, there is limited research on how to best support guideline implementation in kindergartens [13]. With knowledge of potential barriers to and facilitators of implementation of the national guidelines, interventions with tailored strategies could be designed to tackle identified barriers and enhance implementation [14].

There is limited research on the implementation and key implementation determinants (barriers and facilitators) of interventions in kindergarten settings. Public health implementation research in nonclinical community settings is emerging, and some previous researchers [8, 15–22] have explored the implementation of nutrition-related interventions in kindergartens and identified factors influencing their implementation. However, research in this field remains sparse [21, 23, 24]. Roland and Ertesvåg [23] described general implementation barriers in kindergartens, such as not knowing how to translate research into practice, staff with diverse educational backgrounds, knowledge, and experience, poor intervention delivery, and the lack of a positive staff culture. A theoretical underpinning in implementation research enables the identification of barriers and facilitators that may influence implementation success [14]. With this knowledge, one can build on what is known and learn from past mistakes and successes to further adapt existing and develop and implement better implementation interventions with strategies to achieve more successful implementation [14, 25]. Therefore, this study aimed to explore kindergarten staff members’ experiences with a nutrition education implementation intervention and to identify barriers to and facilitators of its implementation, as guided by the consolidated framework for implementation research (CFIR).

## Methods

### Study design

The study was conducted among kindergarten staff in a municipality in Eastern Norway. A clinical nutritionist designed and implemented a nutrition education implementation intervention in kindergartens to enhance the implementation of the Norwegian guidelines. The implementation intervention was a one-time PowerPoint presentation about the national guidelines for food and meals in kindergartens, the dietary guidelines from Norwegian Directorate for Health (NDH), trend diets and authorities in media, children who eat a lot and children who eat little, and allergy and food intolerance. This implementation intervention was aimed at all kindergarten staff members in the municipality, and the intervention was identical in all presentations. Sixteen of the municipality’s nineteen kindergartens received the implementation intervention between 2019 and 2021, the strategy of which could be defined as an educational outreach visit. The implementer (the clinical nutritionist) met with the providers (kindergarten staff) in their practice settings to educate them about the intervention (nutritional guidelines), with the intent of changing the providers’ practices [26]. Here, the nutrition education implementation intervention will be referred to both as “the implementation intervention” and “the presentation,” and the nutritionist will be referred to as the “lecturer,” since this was how these terms were used in the interviews. Notably, for an implementation intervention to reach its goals, it is important to identify the barriers to and facilitators of its implementation [27]. The current study used a qualitative study design with individual interviews and was conducted in line with the COREQ checklist [28].

### Theoretical underpinnings

CFIR is meta-theoretical and comprises common constructs from existing theories within five major domains: intervention characteristics, outer setting, inner setting, characteristics of the individuals involved, and the process of implementation [29]. The research group identified this framework as the most relevant for this study based on its wide application across different contexts [30], its comprehensiveness, flexibility, and clear and consistent language and terminology, and the possibility of comparison with identified determinants in similar studies [29]. Furthermore, the possibility of applying the framework post-implementation to inform adaptations of interventions and implementation [30] was considered valuable for this study.

### Participants and recruitment

Kindergarten staff (*n* = 12) from five kindergartens in Nes municipality participated in individual interviews. Participants were purposively recruited based on their attendance at the presentation [31]. Three of the five participating kindergartens were private, and two were public. We aimed to interview kindergarten staff members in different positions (education leaders, kindergarten teachers, child and youth workers, and assistants) to investigate multiple nuances of their experiences and perceptions [32]. Participants were recruited from both private and public kindergartens for heterogeneity purposes.

A clinical nutritionist provided a list of the kindergartens receiving the presentation and the managers’ contact details. The kindergarten coordinator in the municipality and the clinical nutritionist informed the kindergarten managers about the study in advance. To choose which kindergarten to contact, the kindergartens were given numbers. Each number was written on a piece of paper and randomly chosen. Subsequently, the managers were contacted by e-mail and later by phone to ask about talking with a diverse group of kindergarten staff members. The managers shared the contact details of interested staff, who were contacted and received written information about the study.

Participants were given the opportunity to attend the interview in person at their kindergarten, or on Zoom. Participants gave their written consent for in-person interviews and oral consent on audio for Zoom interviews in advance [33]. All participants were assured of confidentiality and the opportunity to withdraw from the study at any time, both written and oral.

### Data collection

A semi-structured interview guide was developed by the authors for the purpose of this study (Supplementary file 1). The domains of CFIR [29] built the basis for the themes in the interview guide. The themes were further developed by discussion of their relevance with the research group and the clinical nutritionist in the municipality. A pilot test was conducted by the first author (an MSc in public health nutrition) with a kindergarten manager in person, which led to minor changes in wording and suggestions for follow-up questions. A question about the length of the presentation was added. The interview guide worked well, and the pilot interview was included in the final analysis. Twelve interviews were conducted by the first author in October–December 2021. The interviews provided extremely rich data, and the information’s power was considered high [34]. The data were collected during the Covid pandemic, so all interviews except the pilot were conducted on Zoom. The Zoom interviews took place from the participants’ homes or kindergartens. All interviews were audio-recorded and transcribed verbatim.

### Data analysis

The analysis was inspired by reflexive thematic analysis (TA), which was developed by Braun and Clarke [35, 36] and involves six steps. We used an inductive analysis approach to also explore themes that might not be covered by the framework. To secure trustworthiness, the interviewer (CBL) prolonged engagement with the data by spending time with the participants and by mapping the context of the food environment in their kindergartens through conversations with the leaders and the clinical nutritionist who presented the intervention. In step one, two researchers (CBL and LGH) became familiar with the data by rereading the transcripts. Second, CBL generated initial codes in NVivo 12, where everything of interest to the research questions was marked to avoid overlooking potential themes. Codes with similar meanings were then merged into subthemes in step three and identified as either a barrier or a facilitator. Step 1 to 3 were carried out through researcher triangulation, where CBL and LGH discussed the codes and subthemes. We revised the potential subthemes in step four, and codes within each subtheme were first systematically reviewed, followed by rereading the transcripts. In step five, we defined and named the themes. Steps 4 to 5 were carried out by CBL, LGH and BM. Related subthemes were merged into main themes. The transcripts, codes, and themes were discussed by the researcher team until we reached team consensus and revised several times during the analysis to ensure that they comprehensively and concisely captured what was meaningful about the data in terms of the research questions.

## Results

### Kindergarten and participants’ characteristics

Twelve kindergarten staff members across five kindergartens were interviewed, with seven participants representing three private kindergartens and five participants representing two public kindergartens. Seven participants were educational leaders, and three were managers in a kindergarten. Additionally, one participant was a child and youth worker, and one was a kitchen assistant. All the participants had several years of experience working in kindergartens, with most having more than 10 years of experience. The interviews lasted 40–80 min. The kindergarten size, kitchen facilities, meals, and people responsible for food preparation varied among the kindergartens (Supplementary file 2).

The participants were interviewed up to two years following the presentation, and many found it difficult to remember the presentation in terms of time, planning, execution, and content. Therefore, questions to explore why these factors occurred were added to the interview guide. The participants explained that the extensive time since the presentation, a general focus on food and meals, a lot happening in the kindergarten, and not finding the topic relevant or important were reasons why it was difficult to remember.

**Fig. 1:**
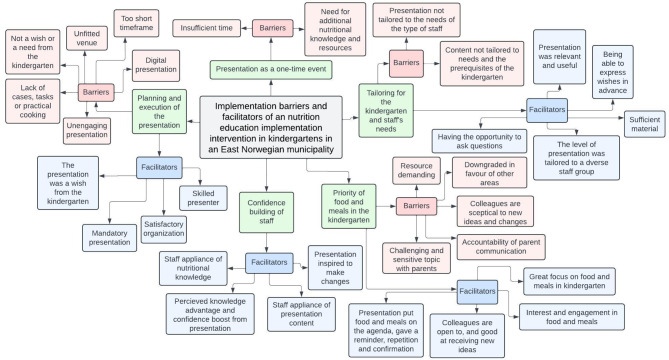
Main themes and sub themes

Figure 1 provides an overview of the main themes and sub-themes. Most of the kindergarten staff members reported that they had attended the presentation at their own kindergarten, in a classroom or meeting room, on a planning day or night, with a smart board, and with all staff in attendance. The participants from one kindergarten said that they received the presentation digitally with other kindergartens due to Covid. Additionally, two participants stated that they attended the presentation at one other kindergarten, and one reported that few kindergarten staff members attended due to the driving distance. In another kindergarten, only three staff members attended because the presentation was scheduled during working hours, when most staff were unavailable.

### Planning and execution of the presentation

The planning and execution of the presentation was identified as a barrier to implementation when it was perceived as unfitting and as a facilitator when it was considered satisfactory. Some of the participants did not consider the presentation a request from the kindergarten but rather from the nutritionist. An education leader stated the following:

[…] it was a wish from her [nutritionist]. But I know it wasn’t something that we in the staff group thought was very import— […] that it was something we requested. There were some things that we wondered about, but it was not anything that was burning out in the kindergartens. (Participant 1)

Others experienced the presentation as a wish or request from within the kindergarten because nutrition is perceived as an important topic. Making the presentation a part of a planning day or night, and thus mandatory, was described as a facilitator of reaching all staff. An education leader explained this as follows:

[…] Because if we send one of us on a course to try to bring it back, it is more difficult. […] with planned nights, I think it’s good that it’s mandatory, and in our kindergarten, the nutrition part is an important part of what we offer as well. So I think it’s important that it’s mandatory, as almost everyone must be able to cook. (Participant 6)

The participants from the digital presentation expressed a lack of contact with the nutritionist, stating that a physical presentation would be more engaging, educational, and memorable. Little information and time for questions were important. While several participants found the length sufficient and appropriate, one education leader said, “The thing about the teaching format, a one-hour meeting, is that you scratch the surface a little […] you never really get in depth” (Participant 8). Additionally, a PowerPoint presentation with pictures was mentioned as a good facilitator to follow compared to listening only. Several participants felt tired and leaned back, finding the nutritionist unengaging and feeling that they were missing a practical component that would help them become more involved and acquire more knowledge. For example, an education leader noted that it would be helpful if “[…] you have to think a little yourself instead of her saying that for breakfast it is wise to serve this and that […] so that you reach those who just sit back and listen” (Participant 2). Others described the nutritionist as skilled with great knowledge and a good presenter, and the presentation as interesting and easy to follow.

### Presentation as a one-time event

Several participants found the one-time presentation insufficient and thought they needed additional nutritional knowledge and resources. The driving distance to the venue and challenges with the venue (e.g., uncomfortable chairs) affected some staff members’ ability to attend and follow the presentation. Regular opportunities to revisit the topic were mentioned by some as desirable. This would allow them to gain more knowledge and have fresh reminders, enable knowledge maintenance, and make and maintain changes within the kindergarten. A more organized follow-up was mentioned: “[…] not just go around and have a presentation one evening and then it stops there […] the content is very useful right there, and if you do not refresh or get a reminder or someone requests anything, it may be a bit off” (Participant 6).

### Tailoring to kindergarten and staff needs

Tailoring was identified as a barrier and a facilitator depending on whether participants experienced the presentation as tailored to the needs of their kindergarten and staff. Several participants missed an opportunity to express their wishes and needs in advance. This led to important topics being missed and the presentation being perceived as irrelevant and unengaging. Thus, contacting the nutritionist when needed was found to be useful because it tailored the presentation to their specific needs. An education leader emphasized that nutritional knowledge was needed:

[…] we know very well what a diet should be like. It’s not, in a way, what limits us. It’s completely different things. So, I don’t know if guiding us on diet does anything other than bring it up [on that] day. Then we think about it, and then maybe we can’t do much about it anyway. (Participant 8)

Others found the presentation relevant and useful, as they felt a general need for nutritional knowledge. Some were able to share their wishes for content in advance, which was perceived as a great opportunity to ensure that no content was overlooked.

The presentation was perceived as aimed at all staff members, regardless of position, knowledge, or interests. However, a few participants described not feeling responsible for parental communication about food and meals in kindergartens because it was the education leader’s responsibility. As a result, a childcare and youth worker (Participant 5) said that they did not know how to proceed in conversations with parents about packed food and preferred not to discuss it with them. Additionally, an education leader (Participant 1) believed that exceptional cases were not within their responsibilities; rather, they should be referred to the nutritionist.

Prior nutritional knowledge was described as depending on one’s position and responsibility in the kindergarten, with education leaders being the most knowledgeable. Some participants perceived the level of content as too general, while others found it too high. This affected their perceptions of the usefulness of the presentation. One education leader explained this as follows:

They [assistants] do not have the same planning time as us, so they do not feel all the responsibility in the same way. There were not many who thought it [the presentation] was that useful because these are matters [that] we, as educators, should know. (Participant 1)

Other participants found the presentation easy to understand and follow for diverse staff, which was described as important since nutrition is essential for all staff. A manager added, “It’s about not making it so complicated too, because then it gets boring and [un]interesting” (Participant 10).

The opportunity to ask questions for guidance specific to their kindergarten made the presentation more tailored and was perceived as important by all participants. An education leader said, “[…] you have different children who can have different problems […], to be able to ask about it, at the time, is in a way very useful” (Participant 6).

### Priority of food and meals in kindergartens

The priority theme concerns the participants’ perceived priority of food and meals in their kindergarten, as well as how they prioritize these areas themselves. They stated that a lack of priority may have been a barrier, and that making these areas a priority could have facilitated the implementation. The participants described food and meals in the kindergarten as resource-demanding, and due to a lack of resources, such as time, the topic was often seen to be less prioritized in tasks and during discussions. The priority of these areas was described by an education leader as follows: “There are so many areas we must take care of in one day […] We have to somehow concentrate sometimes on one, or choose what we will go more in depth on […]” (Participant 6). Additionally, a participant from the kindergarten with only three staff members in attendance explained that the topic would not be prioritized at larger meetings:

I think that if this was a theme that we were going to have at a staff meeting, for example, to the whole staff, we would not have it. […] We do not have time for it. We have so little common time. There is so much else […] So, then this [the presentation] would not have been prioritized at all. (Participant 8)

Other participants explained that the presentation was important and useful because food and meals are a kindergarten priority and part of what they should be working to improve.

The participants also explained that the topic would not be prioritized for discussion with parents since food habits were perceived as slightly sensitive, and parental communication is important. Thus, some found such discussions challenging and uncomfortable. An education leader in a kindergarten in which parents provided breakfast described it as follows: “[…] for us to go and pick too much in it, we really have neither the opportunity nor the desire to […] It does something with the relationship […] you might want to save it for something else” (Participant 8). Some participants described themselves as interested in prioritizing food and meals in their kindergartens, while a few expressed that it was not of interest to them. Some described their colleagues as skeptical of new ideas and change, with negative attitudes toward the presentation: “[…] many people who worked in a kindergarten for a lifetime. Things have always been like this, and therefore [they] shall continue to be like that” (Participant 3). Other colleagues were described as being open to new ideas from outside and as having positive attitudes toward the presentation.

The presentation was described as giving a needed reminder, repeating the ideas, and confirming that putting food and meals on the agenda should be a higher priority. An education leader explained it as follows: “[…] because all the themes are in a way lying there as such a part of the kindergarten soup. And getting things brought to light is always healthy in relation to reflect[ing] a little on one’s own practice” (Participant 8). Another education leader mentioned how, after the presentation, they “try to have more focus on” (Participant 3) the national guidelines for food and meals in kindergarten.

### Confidence building of staff members

The presentation contributed to building the confidence of kindergarten staff members—a facilitator of the presentation’s implementation. The participants explained how they usually referred to nutritional knowledge to justify their points of view in disagreements with parents or colleagues. Several thought that the presentation provided an advantage regarding nutritional knowledge and that they felt more confident about their own knowledge and in conversations with others. An education leader stated, “We have gained a lot of knowledge and important information, so I can feel more confident and also justify my choices—why we do as we do” (Participant 4). Additionally, the presentation was seen as motivating and as a way to influence decisions to make changes in kindergarten practices.

## Discussion

This study identified the barriers to and facilitators of the implementation of a nutrition education implementation intervention. Prioritizing food and meals in the kindergarten, tailoring the presentation to kindergarten and staff needs, and confidence building of staff members through the presentation were identified as important facilitators for successful implementation. Barriers were related to the engagement of the lecturer, physical environment and to remembering the intervention since it was a one-time event.

### The importance of prioritization and time commitment

We identified the kindergarten’s prioritization of nutrition as an important factor for successful implementation of the intervention. Some participants perceived it as a response to kindergarten needs and others as an external wish. This distinction may be crucial for the successful implementation of an intervention [29]. Norwegian kindergartens are obligated to address the topic of nutrition [10]. Thus, the content was perceived as a reminder, a repetition of ideas, and a confirmation rather than novel information. As the participants in our study and Roland and Ertesvåg [23] emphasized, kindergartens have various areas they have to prioritize. These tasks can compete with each other on time and resources [23]. Other studies [15, 20] have identified food and meals as less prioritized when competing tasks arise. Carraway-Stage et al. [15]. identified varying priorities for nutrition at the administrative and individual levels among staff. The priority level of nutrition was directly reflected in the amount of resources and time allocated to it [15]. These findings are consistent with our findings. If nutrition was a priority, then kindergarten staff would dedicate more time and effort in trying to give input to an intervention such as the presentation that was the topic of this article. This would mean that the content of the presentation as well as the delivery would be tailored to the needs of the kindergarten staff and thus, repetitiveness would not be a barrier. Hence, the prioritisation of nutrition in the kindergarten is important to increase the successful implementation of national guidelines.

As identified in our interviews, lack of time is a well-known barrier to the implementation of kindergarten interventions [8, 18, 20–23] and is captured by the CFIR construct of available resources [29]. Time-related challenges for kindergarten staff are particularly prominent considering how many different tasks they have, and the variety of different areas on which they must focus. Thus, if nutrition was given priority as already discussed, it would also be more justifiable for staff to spend some of their valuable time on this task, as opposed to others. Time, combined with lack of prioritization are important barriers influencing the successful implementation of the presentation.

### The presentation as a confidence booster for kindergarten staff

Nutritional knowledge often served as a tool for resolving disputes with parents or colleagues. This could have been a facilitator, as it might have enhanced the perception of the usefulness and relevance of the implementation intervention. However, discussions on nutrition with parents were not prioritized, reflecting its sensitive nature [19, 37] and the complexity of parental involvement in nutrition interventions [8, 18, 19, 22]. The participants perceived the presentation as important and useful when it was compatible with the perceived priorities and needs of the kindergarten, while the opposite was also seen. This is captured by the CFIR construct of relative priority [29]. Additionally, some participants felt that the presentation itself put nutrition on the agenda. Ongoing support has been identified as important, as it reminds and helps motivate participants to implement and sustain health-related changes [8]. As mentioned, this could be facilitated through regular short sessions with external follow-ups. The nutrition education implementation intervention boosted the participants’ confidence and self-efficacy in nutrition discussions and actions, potentially facilitating implementation. Staff beliefs in their implementation abilities are captured by the CFIR construct of self-efficacy [29]. While this study did not explore how staff members applied new knowledge, affirming existing knowledge might be an important implementation facilitator for national guidelines in kindergartens.

### The importance of tailoring

Our intervention was a one-day event and several participants did not remember the content due to this short duration. Similarly, kindergarten staff members who attended training as a part of the Healthy Start–Départ Santé intervention experienced the training as too short to go in depth and cover important topics; thus, it was perceived as an implementation barrier [8]. A one-time presentation was perceived as insufficient for several participants in our study, both in terms of additional knowledge and maintenance. Swindle et al. [21]. highlighted the demand for more frequent but brief interactions [21]. The length and frequency of the implementation intervention are captured by the CFIR construct design quality and the packaging of the intervention [29]. Regular, short sessions with external follow-ups could make nutrition a priority without it being overwhelming, considering the time pressures of staff, and foster sustained change to potentially alter kindergarten culture.

Despite the potential for a tailored and adapted implementation intervention for the needs of the different kindergartens, the uniformity of the provided presentation and the lack of pre-engagement with staff members’ needs led to perceptions of irrelevance and disengagement. Researchers have emphasized the involvement of end users from the beginning through a more participatory health research approach as a significant implementation facilitator [16]. Involving those understanding the context in the pre-implementation phase could reveal factors that might have been unknown to the intervention developer and implementer [25]. Several researchers [11, 16, 23] have emphasized the importance of this local adaptation. Based on knowledge of local contexts, such as individual attributes, including individual perceived needs, nutritional knowledge, responsibility, interest, attitude, and organizational needs, as well as the priority of the different kindergartens, local adaptations can enhance implementation quality and be a better fit for each kindergarten [23]. This is captured by the CFIR construct of adaptability and compatibility [29]. This finding is consistent with other studies that have reported compatibility as an important factor in the implementation of interventions in kindergartens [38]. A tailored presentation to the local context and setting would have ensured that participants found the content valuable and the time dedicated worth their effort, ensuring a smoother implementation process addressing their specific needs.

### The engaging lecturer and the logistics of delivery

The engagement level was related to the content not being compatible with perceived needs, lack of engagement from the lecturer, lack of a practical component, and the mode of delivery (digital vs. in-person). These determinants were perceived as important for knowledge acquisition of the nutrition education implementation intervention. Similarly, Van De Kolk et al. and Roland and Ertesvåg described the enthusiasm of the implementer [22] and delivery quality [23] as important implementation determinants. The nutritionist’s ability to engage the kindergarten staff is captured by the CFIR construct of execution [29]. Several researchers have emphasized the importance and benefits of interactive learning in implementation trainings [16, 21, 39]. This increases the likelihood of retainment and application of what is being taught [39] and is captured by the design quality and packaging construct of CFIR [29]. Furthermore, those attending the nutrition education implementation intervention digitally found it less engaging and educational. Nesher Shoshan and Wehrt [40] explored such limitations with Zoom, including the reduced richness of social cues in video conferencing as a source of exhaustion and difficulty maintaining concentration [40]. However, the physical learning environment of the implementation intervention we studied was also affected by easily adjustable but overlooked factors, such as uncomfortable chairs. This was perceived as affecting the acquired knowledge. CFIR’s planning construct highlights the importance of incorporating stakeholder needs, suggesting the need to involve kindergarten staff early in the planning stage [29].

### Limitations and strengths

Kindergarten managers were the main contacts for recruitment and could not share staff members’ contact details due to privacy concerns. This might have been a limitation, as recruitment relied on the managers’ engagement in the study. The interviews were conducted up to two years after the implementation intervention. Managers and participants explained that the low participation response was related to struggling to remember the presentation and its content, sickness among the staff, and staff not wanting to do “extra work.” The participants were mainly educational leaders with several years of experience. Staff with other positions and less experience might have had other experiences with the presentation and its usefulness. Even though we have taken several considerations to secure the trustworthiness of the data throughout the research process (e.g. researcher triangulation and team consensus in the analysis) our findings cannot be transferred to all the kindergartens who received the same intervention [41]. However, our detailed description of the participants’ experiences in the result section, may allow those who seek to transfer the findings to their own experiences to judge the transferability to the experiences in their kindergarten.

Our research approach of using CFIR to design data collection and reflexive TA for analysis helped ensure that possible barriers and facilitators known to influence implementation were explored and questioned during data collection.

## Conclusions

This study shows the importance of considering the following factors for the successful implementation of short interventions to support healthy eating in kindergartens:

- Facilitate a participatory approach in the design/pre-implementation phase, which involves kindergartens and staff members. The purpose is to map individual attributes, identify kindergartens’ needs, and understand their priorities.
- Actively engage kindergarten staff through the performance of the implementer and by introducing practical tasks for staff to acquire the knowledge presented.
- Physical learning environments, such as the time and place of the presentation, chairs, and the ability to see the presentation, are important.
- A higher frequency of short presentations are opportunities to revisit the topic and conduct external follow-up while considering limited time resources.

Future research should investigate how nutrition implementation interventions can be tailored towards the kindergartens’ specific needs.

### Electronic supplementary material

Below is the link to the electronic supplementary material.

Supplementary Material 1

Supplementary Material 2

## Data Availability

The data analysis for this manuscript can be made available upon reasonable request by contacting the corresponding author.
