# Supplementary material for: Implementing a nutrition education intervention in Eastern Norwegian Kindergartens: barriers and facilitators
Source: BMC Nutr. 2024 Jul 24;10:103. doi: 10.1186/s40795-024-00908-z (PMC11270914; doi:10.1186/s40795-024-00908-z)
Supplement: Supplementary file 1 — Supplementary Material 1 [file 40795_2024_908_MOESM1_ESM.docx]

Supplementary file 1 Kindergarten and food provision characteristics

| **Kindergarten** | **1** | **2** | **3** | **4** | **5** |
| --- | --- | --- | --- | --- | --- |
| **Participants** | **Pilot, 4, 5, 6** | **1, 2** | **3,** | **7, 8** | **9, 10, 11** |
| **Ownership** | Private | Private | Private | Public | Public |
| **Children in total** | Approx. 50 | Approx. 60 | Approx. 90 | Approx. 60 | Approx. 100 |
| **Kitchen facilities** | 2 kitchens. Not institutional. | 1 big kitchen for hot meals and 4 smaller ones. Not institutional. | 3 kitchens. Not institutional. | 1 kitchen. Not institutional. | 1 big institutional for kitchen assistant. 4 smaller ones for other staff. |
| **Meals in the kindergarten** | All meals served.  One hot meal every day. | All meals served.  One hot meal a week. | All meals served.  One hot meal a day. | Parents provide packed food for breakfast. Kindergarten provides 2 meals.  One hot meal a week. | All meals served  Hot meals 2-3 times a week. |
| **Responsibility of food preparation** | All staff. Early shift or those with interests. | All staff. Usually early shift. | All staff. Usually early shift. | All staff.  Usually those with interests. | Kitchen assistant for hot meals. Usually early shifts other days. |
