# Supplementary material for: Implementing a nutrition education intervention in Eastern Norwegian Kindergartens: barriers and facilitators
Source: BMC Nutr. 2024 Jul 24;10:103. doi: 10.1186/s40795-024-00908-z (PMC11270914; doi:10.1186/s40795-024-00908-z)
Supplement: Supplementary file 2 — Supplementary Material 2 [file 40795_2024_908_MOESM2_ESM.docx]

**Interview guide**

**Introduction**

- About the project

- The purpose of the interviews

- Practical information (audio recordings, confidentiality and consent)

**Introduction to the topic – General**

- Can you describe the role meals and diet play in your kindergarten?
- Can you give some examples of meal situations?
- Can you describe any other issues/areas (other than food/meal related) that are important to your kindergarten?

**About the course** about healthy eating that you have had in your kindergarten

Can you describe the organization of the course?

- What time of day did you receive the lessons?
- What did the rest of the day look like? Can you describe the course of the day?
- Who was present at the class?
- What do you think about the organization of the course?

What do you think about the content of the course?

- Can you give examples of some of the content you found helpful?
- What was the amount of information like?
- Do you remember if you felt something was missing?

Have you discussed the course with any colleagues?

- If so: can you give me some examples of what was discussed?
- In what context did these discussions occur? (formal or informal)
- How are new ideas brought up in your kindergarten?
- Can you give me an example and take me through the process?
- Have you discussed the course with anyone outside the kindergarten?

**Implementation of the content of the course**

Can you tell me about your experiences communicating with parents about food and meals in kindergarten before class?

- How do you perceive the need to strengthen this communication?
- Have you used the content of the course in communication with parents?
- If yes: Can you give examples of how it has been used?
- Have there been any changes in communication with parents? In what way?
- What are the parents' reactions?
- If no: What do you think might be the reason you don't have?
- In what way do you think that applying what you have learned in the course can affect communication with parents?
- Can you give me some examples of how you can use what you have learned in conversation with parents about food and meals in kindergarten?
- To what extent do you feel confident trying new things to improve how you do your tasks at work?
- Does your kindergarten have a person who has special responsibility for this communication?
- How do you feel about applying what you've learned when communicating with parents?
- Which part(s) of communication are you most confident in?
- What do you still feel unsure about?
- Can you give me examples?
- How has this teaching affected the use of the food and meals guidelines in kindergarten?
- Have the guidelines become a topic of discussion among staff after teaching?
- Can you give me some examples of what has been discussed?

Do you find that there have been changes in the follow-up of individual children after the teaching (small eaters or big eaters)?

• In what way? Can you give me some examples?

• How do you feel about using what you have learned in the course when following up or adapting for children with special needs?

Do you have access to any materials that you can rely on when communicating with your parents about food and meals?

- If yes:

Where can you find it? Easy/hard to find? Easy/difficult to use?

- How do you use these supporting materials?
- Can you give me a recent example where you used some of the content in communicating with parents?
- Do you feel that is sufficient?
- If no: what do you feel is missing?
- If no:

Is there a need for this? What do you feel is missing?

- What kind of support have you received from management in connection with communication with parents about food and meals in kindergarten?
- General/before intervention?
- When using the content of the course?
- Have the managers made arrangements to discuss the teaching and use of the content afterwards?
- If yes: How was this organized? (formal vs. informal); How are shared experiences and opinions received?
- If no: Is this something you think could have been helpful? In what way?
- To what extent do you think that using the content from the teaching will be a priority in your kindergarten?
- Give examples of what is given higher priority.

Suggestions for improvement

- Do you have any further suggestions on what changes can be made to improve the course or use of the content?
- Would you like to learn about children's diets in kindergarten in a different way than through teaching?
- Can you give me an example? (guidance in cases concerning anonymised individual children, posts to parent meetings, practical cooking)
- Is there another existing teaching/material/strategies/training or anything else you've heard of that you think would be a better fit?

Summarizing

- Is there something that you want to add?
